# Working to improve the management of sarcoma patients across Europe: a policy checklist

**DOI:** 10.1186/s12885-018-4320-y

**Published:** 2018-04-16

**Authors:** Bernd Kasper, Estelle Lecointe-Artzner, Suzanne Wait, Shannon Boldon, Roger Wilson, Alessandro Gronchi, Claudia Valverde, Mikael Eriksson, Sarah Dumont, Nora Drove, Athanasia Kanli, Markus Wartenberg

**Affiliations:** 1 University of Heidelberg, Mannheim University Medical Center, Interdisciplinary Tumor Center, Sarcoma Unit, Theodor-Kutzer-Ufer 1-3, D-68167 Mannheim, Germany; 2 InfoSarcomes and Sarcoma Patients EuroNet (SPAEN), 6 rue de la Tremblaie, F- 35510 Cesson-Sévigné, France; 3 The Health Policy Partnership, 68-69 St Martin’s Lane, London, WC2N 4JS UK; 4 Sarcoma UK, 49-51 East Road, London, N1 6AH UK; 5 Department of Surgery, Fondazione IRCCS Istituto Nazionale dei Tumori, Via Venzian 1, 20133 Milan, Italy; 6 Oncology Department, Vall d’hebron University Hospital, Pg Vall d’Hebron, 119-129 Barcelona, Spain; 7 Department of Oncology, Skane University Hospital and Lund University, 221 00 Lund, Sweden; 8 Medical Oncology Department, Gustave Roussy Cancer Campus, 114 rue Edouard Vaillant, 94800 Vilejuif, France; 9 Eli Lilly & company, Avda. de la Industria, 30, 28108 Alcobendas, Spain; 10 Currently at Varian, formerly Eli Lilly & company, Papendorpseweg, 83 3528 BJ Utrecht, The Netherlands; 11 Sarcoma Patients EuroNet (SPAEN) & Das Lebenshaus e.V, Das Wissenshaus GmbH, Untergasse 36, D-61200 Wölfersheim, Germany

**Keywords:** Sarcoma, Rare cancer, Policy, Cancer reference Centre, Multidisciplinary team, Europe, Access, Care pathways

## Abstract

**Background:**

The Sarcoma Policy Checklist was created by a multidisciplinary expert group to provide policymakers with priority areas to improve care for sarcoma patients.

**Main body:**

This paper draws on this research, by looking more closely at how France, Germany, Italy, Spain, Sweden and the United Kingdom are addressing each of these priority areas. It aims to highlight key gaps in research, policy and practice, as well as ongoing initiatives that may impact the future care of sarcoma patients in different European countries. A pragmatic review of the published and web-based literature was undertaken. Telephone interviews were conducted in each country with clinical and patient experts to substantiate findings. Research findings were discussed within the expert group and developed into five core policy recommendations. The five identified priority areas were: the development of designated and accredited centres of reference; more professional training; multidisciplinary care; greater incentives for research and innovation; and more rapid access to effective treatments. Most of the countries studied have ongoing initiatives addressing many of these priorities; however, many are in early stages of development, or require additional funding and resources.

**Conclusion:**

Gaps in access to quality care are particularly concerning in many of Europe’s lower-resourced countries. Equitable access to information, clinical trials, innovative treatments and quality specialist care should be available to all sarcoma patients. Achieving this across Europe will require close collaboration between all stakeholders at both the national and European level.

## Background

### What are sarcomas?

Sarcomas are a family of rare cancers with a combined incidence of six people per 100,000 population, with 28,000 new cases each year in Europe [1]. They develop in the connective tissues and bones, and can occur anywhere in the body, at any age [1]. There are more than 50 subtypes of soft tissue sarcoma (STS) alone, each with unique clinical, prognostic and treatment characteristics [1, 2].

Because of this heterogeneity, sarcoma epitomises many of the challenges encountered with rare cancers. Patients often lack access to appropriate information about their condition, specialised care centres, appropriate courses of care, and ongoing clinical trials [3]. The heterogeneity of sarcoma also complicates research efforts: recruiting sufficient numbers of patients to large clinical trials is often impossible, epidemiological data are scarce and the evidence base to guide clinical practice is inadequate [1, 4, 5].

Sarcoma patients report some of the poorest experiences of any cancer type [3]. Lack of professional experience in diagnosing or treating sarcoma is a critical issue, and may lead to delays and errors in diagnosis and treatment. In one study, 27% of all patients with sarcoma had been told by their general practitioner that their symptoms were not serious, or had been initially treated for something other than sarcoma [3]. Referral pathways are often not clearly defined. Access to appropriate care also varies considerably both between and within countries. Some treatments are not reimbursed, leaving patients to pay for treatments out-of-pocket. Many patients may have to travel great distances to receive appropriate care [4].

### The policy landscape

In the past few years, many stakeholders have raised awareness of the need to improve the patient experience for patients with rare cancers [4, 5]. Sarcoma has been a central focus of these efforts, with publications by the European CanCer Organisation (ECCO) outlining a list of essential requirements for quality care in STS in adults and bone sarcoma [6]; a paper on patient pathways by Sarcoma PAtients EuroNet (SPAEN) defining patient-driven recommendations for sarcoma care [7]; and the creation of a European Reference Network (ERN) on rare adult cancers (EURACAN) including a domain for adult sarcomas [8].

### The sarcoma policy checklist

Building on these initiatives, the Sarcoma Policy Checklist [9] was created and launched at the European Parliament by a multi-stakeholder group in February 2017. The Checklist aims to provide policymakers with priority areas where the greatest needs exist to improve care for sarcoma patients.

This paper draws on research done to build the Sarcoma Policy Checklist, and looks more closely at how six Western European countries (France, Germany, Italy, Spain, Sweden and the United Kingdom (UK)) are addressing each of the recommendations. Drawing from the experience in these countries, the paper highlights key gaps in research, policy and practice, as well as important developments that may impact future policy and practice across Europe – recognising that challenges will differ from one country to another, particularly in lesser-resourced countries in Europe.

A review of the published and web-based literature on sarcoma and rare cancers was undertaken, using standard search terms, which were translated into French, German, Italian and Spanish to enable local language searches in relevant countries. Local language searches in Swedish were deemed unnecessary given that most of the literature is available in English. Telephone interviews were conducted in each country with local clinical and patient experts who are members of the expert group of the Sarcoma Policy Checklist. These helped fill gaps in information and obtain an up-to-date representation of the current situation in each country.

## Recommendations of the sarcoma policy checklist

Research findings were discussed at several stages within the expert group, to ensure consensus in recommendations. The expert group agreed on five key areas where policymakers should focus efforts to improve care for sarcoma patients (the Sarcoma Policy Checklist) and issued recommendations for each of these areas. The Checklist was launched at the European Parliament in February 2017. Recommendations are presented in Table 1.

**Table 1:** The Sarcoma Policy Checklist recommendations, published in February 2017

Recommendations	What is needed in each country?
i. Each country should have at least one designated and accredited centre of reference for sarcoma	• At least one national centre of reference, or a clear link to a centre of reference in another country • National accreditation processes that designate centres of reference using specific quality standards • Regular evaluation of centres of reference against these standards to ensure continuous quality of care
ii. All healthcare professionals involved in sarcoma care should have access to specialised training	• Training on rare cancers included in the general medical curriculum • Specialised training programmes on sarcoma for all healthcare professionals involved in the sarcoma multidisciplinary care team • National referral protocols for suspected sarcoma patients, which advise non-specialists of ‘red flag’ symptoms and when to refer patients to centres of reference
iii. Sarcoma patients should receive multidisciplinary care delivered by a specialised sarcoma team	• All sarcoma patients treated by multidisciplinary teams (MDTs) according to a clear care pathway • A clear definition of the minimum composition of a MDT • National guidelines for the treatment of all sarcomas (children and adults) • A dedicated key health worker and a personalised care plan for each patient
iv. There should be greater incentives for research and innovation in sarcoma care	• Incentives for public–private partnerships • National and international research collaborations for sarcoma • A national sarcoma registry with a standardised dataset to allow comparable real-world data collection across centres of reference
v. Sarcoma patients should have more rapid access to effective treatments	• Alignment between regulatory and reimbursement/HTA agencies on evidentiary requirements for sarcoma treatments • Special regulatory and access pathways for rare cancers • Involvement of sarcoma patients or their representatives in health technology assessment (HTA) and other access pathways • Publicly available national and international clinical trial portals listing all ongoing clinical trials in sarcoma

## Results

Research findings are summarised below based on data from France, Germany, Italy, Spain, Sweden and the UK. This section aims to outline how well each country is doing in meeting recommendations of the Sarcoma Policy Checklist, focusing on some of the most salient issues discussed during expert interviews and identified in our review of the literature.

### Specialist sarcoma care

Centres of reference for the specialised management of sarcomas exist in all six countries, but they are not always formally designated by explicit quality criteria, nor formally recognised by national bodies. The establishment of EURACAN has required countries to nominate centres of reference to participate based on set criteria, formalising the designation of centres for the first time in many countries. In Spain, for example, the Ministry of Health recently endorsed five national sarcoma reference centres to take part in EURACAN [10]. In Germany, two centres – Essen and Mannheim – cover the sarcoma domain within EURACAN. This situation may vary considerably between countries however.

France has an advanced network that connects all clinical and pathological reference centres for sarcoma. There are two clinical networks: French Clinical Reference Network for soft tissue and visceral sarcomas (NetSarc) and French Reference Network for bone sarcoma and rare bone tumours (ResOs) for the medical oncology part [11, 12] as well as a Sarcoma Pathological Reference Network (RRePS), which enables a second expert pathological review for all STS cases to confirm diagnosis [12, 13]. However, a particular issue in France is that the accreditation of reference centres is based on a centre’s expertise in oncology, not surgery. Therefore, the quality of sarcoma surgery varies considerably among centres. This is not necessarily the case in other countries – for example, quality standards in the UK are based primarily on a centre’s surgical expertise.

Even if reference centres are initially designated based on their adherence to specific criteria, their performance over time, in terms of meeting quality standards for surgery, radiotherapy, chemotherapy and other aspects of care, still needs to be regularly monitored [4]. Sweden has a particularly sophisticated monitoring of quality of sarcoma care through its cancer registry for extremity and trunk wall sarcoma [14]. In some countries, such as Italy, there are ongoing efforts by professional societies to clarify quality standards for specific aspects of care (e.g. surgery). In Germany, a formal certification process for reference centres for STS is currently being developed by the German Cancer Society (DKG).

### Professional training

In all six countries studied, there is no formal training on rare cancers (including sarcoma) within the general medical curriculum. Training on rare cancers is also not part of the formal training of oncologists in most countries, although there are ongoing efforts to change this, for example in France and Italy.

Specialist training programmes have particularly focused on surgery at the European level. For example, there is the European School of Soft Tissue Sarcoma led by the European Society of Surgical Oncology (ESSO) [15] and the eSurge programme led by hospitals in both France and Italy [16].

Other efforts exist which target non-specialists to encourage them to quickly refer patients to appropriate specialists and reference centres. For example, the ‘On the ball’ public awareness campaign led by Sarcoma UK (a patient-led charity) aims to raise awareness among general practitioners (GPs) of the ‘red flag’ symptoms of sarcoma, and encourage GPs to refer any suspected sarcoma case directly to specialist centres quickly [17].

Many patient organisations have called for the establishment of clear referral pathways to improve access to expert diagnosis and treatment for sarcoma [7]. Simple referral guidelines, like those for STS in Sweden (which recommend referral to reference centres before initial surgery is performed) have been shown to improve referral rates, reduce costs associated with local recurrence and result in better surgical results and overall patient outcomes [18, 19]. Of patients with deep sarcoma in Sweden, 80% are referred to a regional reference centre before biopsy [20].

Clear referral pathways may help improve the accuracy of diagnosis in sarcoma, which remains an area for improvement. Data from Germany, for example, suggest that the error rate of primary diagnosis is over 60% among non-specialised pathology departments [21], while in France over 45% of first histological diagnoses were modified after a second reading in the French reference networks and possibly resulted in an alternative treatment course [22]. Similar results were found in other regions in France and Italy [23].

### Multidisciplinary care

Most national guidelines, as well as the recent ECCO and SPAEN recommendations [6, 7], recognise that the organisation of sarcoma care in multidisciplinary teams (MDTs) is key to providing high-quality care. This is also a criterion for reference centres to be considered for inclusion into EURACAN.

The networking of centres within both national networks (e.g. NetSarc) and EURACAN may provide the necessary basis for multidisciplinary care practices to develop. For example, in the UK, a National Ewing Sarcoma Multidisciplinary Team (NEMDT) advisory group meets regularly and brings together experts from around the UK to discuss patient treatment plans and best practice, and to find ways to optimise the patient pathway to improve survival rates for Ewing sarcoma patients [24].

However, the implementation of an MDT approach remains uneven between individual centres. Many centres do not have sufficient resources to implement a systematic MDT approach for sarcoma care, and appropriately trained personnel representing each required specialty are not always available. Moreover, the composition of a specialist sarcoma MDT is often not clearly defined.

It is also critical for MDTs to include primary and community-based providers as well as professionals in hospitals or centres of reference, to ensure high quality of diagnosis and care across the entire care pathway [6].

### Incentives for research and innovation

Across all countries, there remains a need for more basic and translational research on sarcoma – and funding to achieve this. Also, clearer regulatory guidance is needed to encourage the establishment of public–private partnerships to drive research in sarcomas; for example, on the appropriate interaction between academia and industry in these collaborations.

There are, however, several important ongoing research initiatives on sarcoma. Individual countries have led focused research efforts in different areas. In 2013, France had 142 started or ongoing translational studies on rare cancers, 30% of them in the sarcoma networks (NetSarc, RRePS and ResOs), and 70 translational studies completed, 49% of them in the sarcoma networks [13]. In the UK, Sarcoma UK published a survey of sarcoma patients in 2015, which has provided important insights into patient experiences [3]. The charity has also made a strategic commitment to fund £3 million in scientific research by 2020 [25]. In Spain, research is taking place on very rare and ultra-rare sarcomas to determine their burden and improve treatment pathways.

As has been mentioned previously, recruiting sufficient numbers of patients into sarcoma clinical trials remains an ongoing challenge due to the small number of patients with each specific type of sarcoma [3–5]. Real-world data pooled from multiple centres as well as established registries at national and international level are therefore critical to gather sufficient evidence of how treatments work in practice, and guide quality improvement efforts. In Sweden, the National Sarcoma Quality Registry (INCA), which collects sarcoma patient data from all regions, provides an interesting opportunity for real-world data analyses. Discussions are also underway to try to link Swedish sarcoma patient data with data from other Nordic countries, as they all follow a similar data collection template. The development of EURACAN is also likely to play an important role in encouraging the collection of comparable real-world data across different centres. Efforts to develop a standardised data set for the collection of hospitalisation data are already in place.

### Access to treatment and care

Access to appropriate treatment and care is a critical concern for patients with sarcoma, and rare cancers more generally. Patient groups are leading efforts in many countries to try to reduce existing disparities in access to treatments [7]. If treatments are not reimbursed, patients and their families may have to pay for them out-of-pocket, causing a considerable financial burden.

Differences in evidentiary requirements between regulatory authorities (for license approval) and Health Technology Assessment (HTA) or reimbursement bodies (for funding) remain a critical issue for patients’ access to treatment. For example, the European Medicines Agency is increasingly allowing flexibility in drug regulatory pathways for orphan drugs and treatments that address clear unmet needs in given patient populations, such as sarcoma. Smaller trials and adaptive trial designs should be given due consideration for rare cancers, as well as accelerated review, conditional marketing authorisation and adaptive licensing [26–28].

This flexibility, however, is not necessarily matched by reimbursement and HTA agencies in most countries [27]. In theory, special regulatory pathways applicable to orphan drugs apply to sarcoma treatments. However, countries do not have consistent approaches to evaluating orphan drugs – creating uncertainty about the level of evidence needed to obtain regulatory approval. This situation often leads to long delays, or even denial of access to patients [4, 27]. Decentralisation of reimbursement and funding decisions to the regional or local level also contributes to significant inequalities in access to new treatments for patients in many countries. Early access or compassionate use programmes often exist; however, they have not necessarily been applied to sarcoma, with some notable exceptions such as the UK.

Another concern often expressed by sarcoma patients and their representatives is their lack of involvement in reimbursement and funding decisions – for example, in helping to determine what constitutes ‘meaningful benefit’ from a patient perspective in funding and reimbursement decisions.

Finally, awareness and participation in sarcoma clinical trials remains inadequate – especially considering that clinical trials are often the only way patients may access potentially innovative treatments. Evidence suggests that sarcoma patients are often not aware of centres of excellence nor of ongoing clinical trials – and physicians may not be aware of existing clinical trials either [3, 5]. For example, the National Sarcoma Survey (2015) in the UK found that the majority of patients (67%) were not asked whether they wanted to take part in a clinical trial and, if they were, uptake was low (22% participation) [3].

## Discussion

It is important to mention that this paper is based on findings from six relatively wealthy European countries. The situation may be very different in some of Europe’s lower-income countries, where lack of available expertise and low levels of resources often result in limited access to quality care for many patients. More extensive research is required to understand the situation in lower-income countries in Europe.

In the six countries studied, there are numerous promising developments in sarcoma; however, many gaps remain. Efforts to improve access to quality sarcoma care and subsequent patient outcomes are still needed, as inconsistent availability of specialist expertise and appropriate referral patterns often result in misdiagnosis and inappropriate treatment for many patients. Strengthening cross-border healthcare initiatives may help, but ideally, every country should have at least one national reference centre for rare cancers with links to more established reference centres in another country. European organisations may also play an important role in building expertise by actively facilitating mentoring, exchange and support programmes to transfer knowledge and practice between countries [7].

Even with one centre of reference in each country and cross-border links between centres, lack of access to quality care may persist within a country. Access will often depend on the existence of suitable transport links. For example, access to a centralised sarcoma reference centre from every part of the country may be achievable in a country with radial transport links, but for countries with varied shape, and distant regions, regional centres for routine treatment would be preferable. That being said, rarer forms of sarcoma would still need to be treated centrally to retain access to true expertise. This may apply, for example, to retroperitoneal sarcomas or complex amputations in the pelvic or shoulder regions. A number of solutions may be envisaged to help improve equity in access to high-quality specialist care across a country, including harmonizing quality criteria for sarcoma reference centres, producing a national treatment strategy and clinical trials portal. Where a centre may lack expertise, technology may also help address knowledge gaps, such as through e-consultations. The growth of IT systems providing decision support software may also allow for more evidence-based decisions even in smaller or less-resourced MDTs.

The certification process of ‘specialist’ sarcoma centres remains a critical issue. While the ideal is for centres to have formal designation with explicit quality criteria and regular quality reviews, this is not usually the case, as mentioned in the paper. The absence of explicit quality criteria is not limited to the field of rare cancers. Self-certification with peer-review would be possible if governments regulated specialised treatment centres, but they generally do not. The role of the ERNs and guidelines in achieving greater harmonisation of quality criteria across designated sarcoma specialist centres remains to be seen.

As has been mentioned previously, the existence of a MDT approach is one of the criteria for centres to become part of the ERN for sarcoma. However, it is important to recognise that consistent implementation of MDTs remains a challenge, as sometimes even specialist sarcoma centres are focused on one aspect of treatment (e.g. surgery) and may lack other specialists to contribute to a comprehensive MDT. Virtual MDTs linking different specialists across centres, again with the help of technology and IT, may offer a potential solution and are being explore in a number of countries, as well as in cross-border care networks. However, it is important to note that having a full MDT at a centralised specialised sarcoma centre is the preference, and technology can act as a tool to facilitate communications between specialists – but not to replace specialists.

Inequities in access result in delays or denial of innovative treatments for patients, effectively limiting their treatment options. Better alignment between evidentiary requirements for regulatory and HTA/access pathways may help reduce delays in access to new treatments for patients.

Low participation in clinical trials remains an important issue for sarcoma patients – often due to low patient awareness of ongoing studies. More patient involvement in clinical trial design is needed to help guide research efforts. Direct patient input into HTA and reimbursement decisions may help ensure value frameworks are closely aligned with patient priorities.

Greater investment in basic, epidemiological, clinical, outcomes and translational research is still needed in sarcoma. Real-world data, aggregated from multiple centres and high-quality registries, will be vital to provide evidence of how effective different interventions are in practice, and guide quality improvement and research efforts as a result. Yet hurdles such as lack of data standardisation need to be overcome before the potential of real-world data can be realised.

Looking beyond national solutions, several pan-European efforts deserve mention and are outlined in Table 2. It is also important to recognise the role of European initiatives aimed at rare cancers more generally.

**Table 2:** European initiatives that focus on improving sarcoma care

Initiative	Links
**The European Organisation for Research and Treatment of Cancer (EORTC) Soft Tissue and Bone Sarcoma Group (STBSG)** develops and coordinates studies on all aspects of sarcomas within the framework of the EORTC. They have an extensive standardised clinical trial database used for various other research projects. Currently, there are 54 member institutions from 14 countries in the group [38].	http://www.eortc.org/research_field/soft-tissue-bone/
**European CanCer Organisation (ECCO) Essential Requirements for Quality Cancer Care: Soft Tissue Sarcoma in Adults and Bone Sarcoma (2017).** They provide oncology teams, patients, policymakers and managers an overview of the elements needed in any healthcare system to provide high-quality care throughout the sarcoma patient’s journey [6].	http://www.croh-online.com/article/S1040-8428(16)30361-4/fulltext
**Sarcoma PAtients EuroNet (SPAEN) policy paper: Sarcoma Patient Pathway Analysis and recommendations for Service Development (2016).** This paper outlines what patients expect sarcoma treatment to look like, how they expect services to be structured and developed to respond to patient needs, and how referral practices should evolve internationally [7].	http://www.sarcoma-patients.eu/en/dings/19-spaen-policy-paper-on-quality-care-in-sarcomas-now-available
**EURO EWING consortium – international clinical trials to improve survival from Ewing sarcoma (EEC project) (2014–2018)**. This initiative is coordinated by University College London and involves 20 European partners. It is funded by the European Union’s Framework Programme 7 (FP7). It is a coalition of clinical study groups to bring greater patient access to clinical trials, promote sharing of knowledge and improve patient results [39].	http://www.euroewing.eu
**euroSARC project**. Funded by the European Union’s Framework Programme 7 (FP7), it aims to design, structure and implement European clinical trials on rare sarcoma subtypes within an integrated translational study network [40].	http://eurosarc.eu/
**Conticabase – European sarcoma database and tumour bank.** The database currently stores information on 16,689 sarcoma patients in Europe, including tumour characteristics, treatment and follow-up, as well as tumour samples [41].	https://conticabase.sarcomabcb.org/

For example, the European Commission-funded RARECARE project, which later evolved into RARECARENet [29], has provided important epidemiological insights into rare cancers [1, 30]. EURACAN, mentioned previously, may significantly improve the quality of sarcoma diagnosis, care, research, and access to clinical trials [8, 31], as well as facilitating cross-border care [32]. However, adequate funding for reference centres still needs to be secured [33].

The Joint Action for Rare Cancers (JARC), of the third EU Health Programme 2014–2020, is also important. It is a collaboration between 18 member states that aims to integrate the needs for rare cancers into national cancer plans by advancing quality of care and research on rare cancers [34, 35]. The JARC provides direct support to EURACAN with implementation, in terms of operational solutions and professional guidance in quality of care, epidemiology, research and innovation, and education [35, 36].

Finally, Rare Cancers Europe (RCE) is leading policy campaigns and actions on many of the key priority areas identified in the Sarcoma Policy Checklist, including: improving patients’ involvement in clinical trial design and participation in clinical trials; standardising, capturing and merging big data for research purposes; improving access to rare cancer therapies; and improving education on rare cancers [5, 37].

Although many of the above initiatives are still in the conceptual or early phases of implementation, they may have a marked impact on the landscape for sarcoma patients in years to come.

## Conclusions

As demonstrated in this paper, there have been many promising initiatives aiming to improve the care of sarcoma patients in recent years. Yet gaps remain – particularly in Europe’s lesser-resourced countries – and we must ensure all sarcoma patients have access to appropriate information, treatment and care. Patients and their representatives should be included in the planning and evaluation of sarcoma care. A multidisciplinary approach to care should be implemented across all expert centres for sarcoma – and link with community providers as well. National and European training programmes are needed to build specialised expertise in the diagnosis and care of sarcomas for all healthcare professionals involved in sarcoma care. Both European- and national-level collaborations are needed to achieve sustainable and equitable solutions in all countries.

## Abbreviations

DKG: German Cancer Society
ECCO: European CanCer Organisation
EORTC: The European Organisation for Research and Treatment of Cancer
ERN: European Reference Network
ESSO: European Society of Surgical Oncology
EURACAN: European Reference Network on rare adult cancers
FP7: Framework Programme 7
GP: General practitioners
HTA: Health technology assessment
INCA: National Sarcoma Quality Registry
JARC: Joint Action for Rare Cancers
MDT: Multidisciplinary team
NEMDT: National Ewing Sarcoma Multidisciplinary Team
NetSarc: French Clinical Reference Network for soft tissue and visceral sarcomas
RCE: Rare Cancers Europe
ResOs: French Reference Network for bone sarcoma and rare bone tumours
RRePS: French Sarcoma Pathological Reference Network
RRePS: Sarcoma Pathological Reference Network
SPAEN: Sarcoma PAtients EuroNet
STBSG: Soft Tissue and Bone Sarcoma Group
STS: Soft tissue sarcoma
